# *In Vivo* Bioluminescent Imaging (BLI): Noninvasive Visualization and Interrogation of Biological Processes in Living Animals

**DOI:** 10.3390/s110100180

**Published:** 2010-12-28

**Authors:** Dan M. Close, Tingting Xu, Gary S. Sayler, Steven Ripp

**Affiliations:** The Center for Environmental Biotechnology, 676 Dabney Hall, The University of Tennessee, Knoxville, TN 37996, USA; E-Mails: dclose@utk.edu (D.M.C.); txu2@utk.edu (T.X.); sayler@utk.edu (G.S.S.)

**Keywords:** bacterial luciferase, bioluminescent imaging, BLI, firefly luciferase, Lux, Luc

## Abstract

*In vivo* bioluminescent imaging (BLI) is increasingly being utilized as a method for modern biological research. This process, which involves the noninvasive interrogation of living animals using light emitted from luciferase-expressing bioreporter cells, has been applied to study a wide range of biomolecular functions such as gene function, drug discovery and development, cellular trafficking, protein-protein interactions, and especially tumorigenesis, cancer treatment, and disease progression. This article will review the various bioreporter/biosensor integrations of BLI and discuss how BLI is being applied towards a new visual understanding of biological processes within the living organism.

## 1. Introduction

Whole animal bioluminescent imaging (BLI) is progressively becoming more widely applied by investigators from diverse backgrounds because of its low cost, high throughput, and relative ease of operation in visualizing a wide variety of *in vivo* cellular events [1]. The ability to visualize cellular processes or other biological interactions without the requirement for animal subject sacrifice allows for repeated imaging and releases investigators from the constraints of considering their process of interest on a “frame-by-frame” basis using labeled slides. In addition, the ability to continually monitor a single individual reduces the amount of inter-animal variation and can reduce error, leading to higher resolution and less data loss. With continuing advances in the hardware and software required for performing these experiments, it is also becoming easier for researchers with little background in molecular imaging to obtain useful and detailed publication-ready images.

The mainstays of BLI are the light generating luciferase enzymes such as firefly luciferase, Renilla luciferase, Gaussia luciferase, Metridia luciferase, Vargula luciferase, or bacterial luciferase [2–7]. Of these however, the firefly, Renilla, and bacterial luciferases are the most popular for optical imaging. These bioluminescent proteins are gaining preference over their fluorescent counterparts because the lack of endogenous bioluminescent reactions in mammalian tissue allows for near background-free imaging conditions whereas the prevalence of fluorescently active compounds in these tissues can interfere with target resolution upon exposure to the fluorescent excitation wavelengths required for the generation of signal output.

## 2. Common Bioluminescent Reporter Proteins

Firefly luciferase (FLuc) is the best studied of a large number of luminescent proteins to be discovered in insects. The genes utilized in most studies are those from the common North American firefly, *Photinus pyralis* [8]. The FLuc protein catalyzes the oxidation of reduced luciferin in the presence of ATP-Mg^2+^ and oxygen to generate CO_2_, AMP, PP_i_, oxyluciferin, and yellow-green light at a wavelength of 562 nm. This reaction was originally reported to occur with a quantum yield of almost 90% [9], however, advances in detection technology have revealed that it is likely actually closer to 40% [10]. Nonetheless, the sufficiently high quantum yield of this reaction is well suited to use as a reporter with as few as 10^−19^ mol of luciferase (2.4 × 10^5^ molecules) able to produce a light signal capable of being detected [11].

Renilla luciferase (RLuc) undergoes a similar method of action to produce bioluminescence. The gene encoding for this protein was originally isolated from the soft coral *Renilla reniformis* and displays blue-green light at a wavelength of 480 nm, however, additional red-shifted variants have been created as well that luminesce at higher wavelengths to promote increased tissue penetration of the luminescent signal. Regardless of the emission wavelength, the RLuc proteins all catalyze the oxidative decarboxylation of its substrate coelenterazine in the presence of dissolved oxygen and perform this reaction at a quantum yield of 7% [3]. Because of its dissimilar bioluminescent signal and substrate, RLuc is often used simultaneously with FLuc for multiple reporter studies.

Bacterial luciferase (Lux) is distinct in function from FLuc and RLuc. Although the most studied of the Lux-containing species are marine bacteria from the *Vibrio* genus, this bioluminescent strategy is present among many known bacterial phyla, and in all documented examples the basic method of bioluminescent production is the same [12]. The Lux operon is organized in a cassette of five genes (*luxCDABE*) that work together to produce bioluminescence in an autonomous fashion at a wavelength of 490 nm. Lux catalyzes the production of light through oxidation of a long chain fatty aldehyde in the presence of oxygen and reduced riboflavin phosphate. The luciferase is a dimer formed from the *luxA* and *luxB* genes, while the remainder of the genes (*luxCDE*) are responsible for protein products that catalyze production and turnover of the required aldehyde substrate [7]. Because of this self-sufficient design, Lux does not require the addition of a substrate if it is capable of being properly expressed in the host cell.

The advantages and disadvantage of BLI reporter proteins are listed in Table 1.

## 3. Optical Properties of Biological Tissues

The unique constraints of performing data collection from within a living medium must be considered in relation to any choice of reporter system. The detection of a luminescent signal from within a tissue sample is dependent on several factors, including the flux of photons from the reporter, the total number of functional reporter cells in the sample, and the location of the reporter cells within the tissue sample itself [13]. In addition, the visualization of the bioluminescent signal is dependent on the absorption and scattering of that signal prior to detection. One method to control for these conditions is to alter the wavelength of the reporter signal. Increasing the wavelength can both reduce scattering and decrease absorption because the majority of luminescent absorption is the result of interaction of the signal with endogenous chromophoric material. By moving to a more red-shifted emission wavelength, where the levels of absorption within tissue are lower, it becomes possible to measure a greater amount of signal intensity than would be possible from an identical reporter with a lower, more blue-shifted emission wavelength [14]. For this reason, it is important to consider the emission wavelength of a given reporter system, along with the other desired attributes of that reporter, prior to its introduction into any experimental design. For example, the bioluminescent signal from the Lux reaction is produced at 490 nm. This is relatively blue-shifted as compared to the FLuc-based bioluminescent probes that display their peak luminescent signal at 560 nm. The shorter wavelength of the Lux-based signal has a greater chance of becoming attenuated within the tissue and therefore may not be as easily detected if it is used in deeper tissue applications (such as intraperitoneal or intraorganeller injections), and may require longer integration times to achieve the same level of detection as a longer wavelength reporter would when injected subcutaneously. Therefore, if short measurement times and low population level cell detections are the goals of a particular experiment, an FLuc-based reporter would be beneficial compared to a Lux-based reporter despite potential problems introduced through substrate administration in the FLuc system. However, if a near surface detection of large cell populations (such as a subcutaneous tumor) was the end goal, the effects of absorption and scattering could be overcome by the depth and position of the reporter, thus allowing for selection of the more blue-shifted Lux reporter system.

## 4. Imaging Equipment

The challenge of detecting and locating bioluminescent light emissions from within living subjects has been met by several commercial suppliers of *in vivo* imaging equipment (Table 2). A basic imaging system consists of a light-tight imaging chamber into which the subject is placed and a high quantum efficiency charged coupled device (CCD) camera, usually super cooled to less than −80 °C to reduce thermal noise, that collects emitted light. The camera typically first takes a photographic image of the subject followed by a bioluminescent image. When superimposed, regions of bioluminescence become mapped to the subject’s anatomy for pinpoint identification of source emissions. Acquisition times can range from a few seconds to several minutes depending on signal strength. Software displays the image in a pseudo colored format and provides the tools needed to quantify, adjust, calibrate, and background correct the resulting image. Integrated gas anesthesia systems, heated stages, and isolation chambers are typically available to accommodate animal handling.

The technology incorporated into *in vivo* imaging systems is rapidly advancing to meet user needs in a greater diversity of application backgrounds. CCD cameras are being replaced by more sensitive intensified CCD (ICCD) and electron multiplying CCD (EMCCD) cameras that can manage acquisition times of millisecond durations. These fast processing times along with powerful software now permit real-time tracking of conscious, moving subjects (see, for example, the IVIS Kinetic system from Caliper Life Sciences). Anesthesia can have dramatic, unknown, and interfering effects on animals, and the ability to image in its absence is a major step forward in *in vivo* imaging technology. However, these newer imaging systems still remain far too expensive for the typical researcher and to date most imaging is still performed on anesthetized animals. Imaging systems are additionally becoming better integrated with existing medical technologies for multi-parameter analyses. For example, electrocardiogram (ECG), X-ray, or computed tomography (CT) procedures can operate in parallel with imaging acquisition. The ability of software to overlay and map these data to the bioluminescent image offers unique opportunities to visualize physiological status and kinetics.

The major drawback of *in vivo* imaging systems is its limited depth penetration under whole animal imaging conditions. In most cases, using a CCD camera to image luminescent or fluorescent signals at depths beyond a few centimeters produces inconsistent results. Without major advances in imaging sensitivity, either with the camera systems, the internal signal, or almost certainly both in tandem, *in vivo* imaging applications may become limited solely to small animals and the translational leap to humans will never occur. Rather than relying on a camera to visualize the signal externally, it may be feasible and potentially more practical to monitor the signal internally using implantable sensors. Although not yet a viable technology, proof-of-concept microluminometer integrated circuits of only a few square millimeters in size have been developed and validated for bioluminescent signal acquisition [15]. These so-called bioluminescent bioreporter integrated circuits, or BBICs, were specifically designed for capturing the 490 nm bioluminescent light signal emitted by the bacterial Lux proteins, and accommodated on-chip transmitters for wireless data transmission. Effectively interfacing the microluminometers with the luciferase reporter systems, maintaining reporter viability, and implanting the chips would remain challenging, as would the regulatory and safety constraints associated with any human implantation experimental approaches.

## 5. Imaging Modalities

### 5.1. Steady-State Bioluminescent Imaging

The classical hallmark of BLI is steady state imaging, a process whereby bioluminescently tagged cells are imaged over time to determine if light output is increasing or decreasing compared to the initial state. In this type of imaging, either a gain or loss of signal can be the desired result depending on the experimental design. Commonly, bioluminescent cells are injected into an animal model to determine the kinetics of tumorigenesis and growth. The use of BLI as a substitute for mechanical or histological measurement of tumors has increased rapidly in recent years as it does not entail high levels of animal subject sacrifice nor tedious histological analysis, and can overcome the loss of accuracy associated with physical analysis due to the contribution of edema and necrotic centers to overall tumor size [16].

By monitoring tumor growth using BLI, an investigator can track changes within individual animals over time without requiring the subject to be sacrificed. This reduces the amount of intra-animal variability and can improve the detection of significant results. Kim and colleagues have recently demonstrated the effectiveness and resolution of the newest generation of these reporters designed for tumor detection. By injecting codon-optimized FLuc transfected 4T1 mouse mammary tumor cells subcutaneously, they were able to image single bioluminescent cells at a background ratio of 6:1 [17]. This type of resolution will allow researchers to continuously monitor cancer development from a single cell all the way to complete tumor formation.

In the opposite direction, decreases in bioluminescent expression can be used to quickly and efficiently perform drug efficacy screening. The same logistical concerns that have propelled BLI forward as the tool for choice for tumor monitoring are also making it the preferred choice for the screening of new compounds directed at tumor suppression or infection control. In addition, the use of mixed culture or whole animal models can more closely mimic the target microenvironmental conditions that may alter the compound’s activity. As one example, McMillin *et al*. [18] illustrated that high throughput scalable mixed cell cultures with FLuc tagged cancer cells can identify anti-cancer drugs that are specifically effective in the tumor microenvironment early in the discovery pipeline, thereby aiding in their prioritization for further study in ways not previously possible.

### 5.2. Multi-Reporter Bioluminescent Imaging

In a basic experimental design, multi-reporter BLI is performed by simultaneously monitoring for expression of two or more divergent luciferase proteins. This is made possible because all of the characterized luciferase proteins have divergent bioluminescent emission wavelengths. This type of experimental design is especially useful when used to monitor potentially co-dependent, or inter-dependent protein expression such as that expressed during the maintenance of circadian rhythm. Here, the expression of multiple genes can be monitored in real time, without the need to expose cells to potentially influential doses of excitation light wavelengths as would be required for imaging using fluorescent targets [19]. Even when expression of the individual genes of interest is static, sequential imaging of multiple luciferase proteins provides a convenient method for localizing expression profiles of each gene *in vivo* [20].

The work of Audigier and colleagues [21] demonstrates how imaging multiple bioluminescent reporters can be an opportune way to monitor translational dynamics using the function of the fibroblast growth factor two internal ribosomal entry site on neural development as a model. To determine the associated ratios of cap-dependent to cap-independent translation, they cloned the RLuc gene upstream of the site and the FLuc gene downstream. By doing so, they were able to quantify and compare the levels of expression of each reporter protein independently from the same sample, helping to reduce sampling error.

### 5.3. Multi-Component Bioluminescent Imaging

Similar to multi-reporter BLI, multi-component BLI relies on the co-expression of an alternate imaging construct, however, in this case the secondary construct is not itself bioluminescent. Classically, the luminescent emission signal of a substrate amended luciferase protein can be harnessed to act as the excitation signal for an associated fluorescent reporter protein, negating the requirement for treatment with a background stimulating exogenous light source. This process, known as bioluminescence resonance energy transfer (BRET) occurs naturally in the sea pansy *Renilla reniformis* and other marine animals [22], but can be used in research settings to boost the luminescent signal of a bioluminescent reporter, or, more popularly, to determine the interaction of two components of interest within a given system.

A widely known example of the utility of this system was the use of BRET to demonstrate the presence of G protein coupled receptor dimers on the surface of living cells. By tagging a subset of β_2_-adrenergic receptor proteins with RLuc and a subset with the red-shifted variant of green fluorescent protein, YFP, it was possible to detect both a luminescent and fluorescent signal in cells expressing both variants, but no fluorescent signal in cells expressing only YFP [23]. This illustrated the close proximity of the two constructs, since the energy transfer required for excitation of the YFP component can only be performed over very short distances and the lack of endogenous luminescence in the YFP excitation wavelength prevents background fluorescent production.

In some cases, the secondary component is not a fluorescent compound but rather a non-independently functional domain of the luciferase protein itself. These types of constructs are easily created using reporters such as FLuc that have distinct N (NLuc) and C (CLuc) terminal domains joined by a linker region. These types of protein structures lend themselves nicely to separation into distinct components that, when brought together, can form a functional luciferase protein.

First described by Paulmurugan *et al*. [24], this process takes advantage of the lack of a bioluminescent signal in small animal tissue samples. The individual N and C terminal components of the FLuc protein are not capable of producing light independently of one another, however, when they were independently tethered to two proteins known to interact strongly, the researchers were able to demonstrate that bioluminescence could be restored upon substrate amendment. The complementation of a single luciferase protein as opposed to the adjoinment of a luciferase with a fluorescent partner does not require the pair matching of a luciferase/fluorescent reporter with overlapping emission/excitation wavelengths, and can permit co-visualization with other reporters in a single subject to permit multi-localization of groups of proteins.

### 5.4. Bioluminescence as a Supplementary Imaging Technique

As the technology for small animal imaging continues to increase in power and availability, there is an increasing movement towards combining multiple imaging techniques to improve the amount of detail that can be obtained from a single subject. While no single imaging technique can provide an investigator with a comprehensive picture of the system as a whole, the combination of multiple techniques such as computed tomography (CT), positron emission tomography (PET), and BLI can help to “fill in the gaps” left by each approach in a rapid, sequential manner. The development of trimodal fusion proteins that are capable of simultaneously acting as signals for fluorescence, bioluminescence, and PET, and the introduction of combined clinical PET/CT scanners has made it possible to obtain more information from a single animal subject than was previously believed possible [25].

## 6. Substrate Delivery Methods

### 6.1. Required Substrate Amendment

The most common bioluminescent proteins employed as targets for whole animal BLI, FLuc and RLuc, require the injection of a substrate compound in order to produce a bioluminescent signal. FLuc requires the injection of D-luciferin, while RLuc requires the injection of coelenterazine. It is only upon oxidation of these luciferin compounds that light is capable of being produced. The route of substrate injection can have influential effects on the emission of a luminescent signal so, although logistical concerns may be most pertinent to consideration for investigators, the method of injection should be considered in light of the proposed objectives of any study [26].

### 6.2. Intraperitoneal Injection

The convenience of intraperitoneal injection makes it an attractive option for the majority of researchers, however, following this route of injection the substrate must absorb across the peritoneum to reach the target expressing cells. Any variations in this rate of absorption can lead to variations in the resulting luminescent signal and can make reproducibility of results increasingly difficult [27]. In addition, investigator error can lead to injection into the bowel, causing a weak or non-existent luminescent signal that can be confused with a negative result [28]. Predictably, intraperitoneal injection provides lower peak luminescence levels than subcutaneous injection when inducing light production in subcutaneous tumor models, however, it has been found that it can also overestimate tumor size when used to induce luminescence from intraperitoneal or spleen-localized tumors, owing to direct contact between the luciferin and the target luciferase expressing cells [26]. The greater availability of the luciferin to the luciferase containing cells can increase the amount of bioluminescent output by allowing them greater access to the luciferin compound without prior diffusion through non-luciferase containing tissue and increasing the influx of the luciferin compound into the cell due to the resulting increased concentration gradient.

### 6.3. Intravenous Injection

Intravenous injection can be used to systematically profuse a test subject with D-luciferin or coelenterazine and expose multiple tissue locations to the substrate on relatively similar timescales. The systemic profusion of luciferin allows for lower doses to be administered to achieve similar luminescence intensities as would be seen using alternate injection routes [27], however, studies using radio-labeled D-luciferin have indicated that the uptake rate of intravenously injected substrate is actually slower in gastrointestinal organs, pancreas, and spleen than would be achieved using intraperitoneal injection [29]. While the intravenous injection of substrate can quickly perfuse throughout the entire subject, the resulting luminescent signal is of a much shorter duration than would be observed using alternate injection routes [26].

### 6.4. Subcutaneous Injection

Subcutaneous injection can be used as an alternative to intraperitoneal injection while avoiding the signal attenuation shortcomings of the intravenous injection route. It has previously been demonstrated by Bryant *et al*. [30] that repeated subcutaneous injection of luciferin can provide a simple and accurate model for monitoring brain tumor growth in rats. It has also been demonstrated that the repeated subcutaneous injection of D-luciferin or coelenterazine into an animal model results in minimal injection site damage and can provide researchers with bioluminescent signals that correlate well with intraperitoneal substrate injection luminescent profiles, albeit with a longer lag time prior to reaching tumor models in the intraperitoneal space [26].

## 7. BLI Applications

The effectiveness, sensitivity, and sophistication of BLI methods and tools have resulted in an ever broadening inventory of applications. Tables 3 and 4 provide a snapshot of current research and developmental activities using the FLuc, RLuc, and Lux BLI systems and the following sections present brief overviews of selected applications.

### 7.1. Small Animal Models

Small animal models, particularly mice, have become the preferred subjects for optical imaging experiments. The use of a model system such as the mouse allows researchers to look at human-relevant processes in a well documented proxy using equipment that performs similar functions, but is much less expensive and requires less space and resources than those employed within the medical field for human subjects. It also allows the researcher to move away from a cell culture setting where the system of interest is not able to be monitored under the same conditions at it would within the organism as a whole. This allows for the conduct of medically important research that can accelerate the transition to human medical use. One example of a common application of this type of research is the use of a bioluminescently-tagged cancer cell line to track the growth dynamics of the cancer over time and in response to various treatment strategies. Zhang *et al*. [31] have recently demonstrated how these two avenues can be investigated simultaneously by using FLuc-tagged MDA-MB-453 cells. By using a mouse model and injecting FLuc-tagged cancer cells, they were able to simultaneously compare and contrast multiple cancer models and at the same time evaluate the effect of several treatment courses.

Virostko and colleagues [32] have demonstrated the usefulness of using a small animal model for direct measurement of human cells through the profusion of pancreatic Islets into mice expressing luciferase under the control of mouse insulin I promoter. This has allowed them to noninvasively look at changes in luminescent response to β cell mass under baseline and diabetic conditions. These types of medically relevant experiments demonstrate the advantages that can be achieved in a short period of time by using a small animal model rather than human subjects or cell culture.

### 7.2. Tracking Cells

BLI is extremely useful for longitudinal assessment of cell fate *in vivo*. When introduced into a living animal, bioluminescently-labeled cells can be repeatedly and noninvasively imaged over time. The intensity and location of the bioluminescent signal can provide insights into the abundance and spatial distribution of tagged cells in the living subject. In addition to visualizing tumor progression *in vivo* by imaging bioluminescent cancer cells injected into living animals, investigators have employed BLI to monitor the behaviors of stem cells [33–41], the response of immune cells in various diseases [42–45], and the rejection and engraftment of transplanted tissues [46–48].

Stem cell-based therapies hold promise in the treatment of cancer, cardiac disease, brain injury and other diseases. Before moving onto clinical trials, however, the behavior and mechanism of action of transplanted cells must be understood *in vivo*. Whole animal BLI allows repetitive and quantitative measurements of cells of interest, providing useful information on cell survival, proliferation and migration over time in the same living subject. For example, different types of stem cells have been extensively used in cardiac regeneration therapies [34,41,49,50]. Recently, van der Bogt and colleagues compared different stem cell types as candidates for treatment of myocardial infarction [41,50]. They utilized BLI to assess *in vivo* fates of bioluminescently tagged bone marrow mononuclear cells (MNs), mesenchymal stem cells (MSCs), adipose stromal cells (ASCs), and skeletal myoblasts (SkMb) after transplantation into a murine myocardial infarction model. Their results suggested that MNs exhibited higher survival rate than other cell types, along with better heart function. Stem cell researches will continually benefit from BLI as a fast and noninvasive tool to visualize cell fate *in vivo*.

In addition to stem cells, immune cells are also attractive targets in research involving BLI. By labeling cells of interest with constitutively expressed luciferase, investigators are able to visualize target cell population trafficking throughout living subjects and homing to disease sites in response to various stimuli [51,52]. BLI enables monitoring immune effector cells (such as cytotoxic T cells and natural killer T cells) in various malignant diseases including graft-*versus*-host disease, cancer, heart diseases, and neurological diseases [42,43,45,53–55]. As an example, investigators employ BLI to assess the fate of adoptively transferred T cells in tumor-bearing hosts to study tumor immunology and immunotherapy. In a recent study performed by Dobrenkov *et al*. [45], BLI was used to longitudinally track human prostate cancer-specific T lymphocytes in a murine prostate carcinoma model. By labeling tumor-targeted T cells with click beetle red luciferase and tagging tumor cells with RLuc, the authors were able to visualize T cell trafficking and tumor progression in the same animals at the same time. This model demonstrates the application of multi-reporter BLI on adoptive T cell-based immunotherapy and host response. By integrating multiple reporter probes, BLI has the potential to visualize complicated biological events involving multiple components of interest.

### 7.3. Monitoring of Genes

Regulation of gene expression is fundamental in cellular and molecular processes. Since more and more genes have been discovered to be regulated or responsive to various signals during disease progression, BLI has been facilitating the studies of conditional and spatiotemporal expression patterns of endogenous genes in living animals to provide better understandings of what is happening *in vivo* in real time. A common approach to monitor gene expression using BLI is to express a reporter gene (*luc*, for example) from the promoter of the gene of interest to test the expression of a particular gene. Alternatively, expressing the reporter gene under the control of regulatory elements responsive to a certain transcription factor can be used to investigate genes regulated by the same transcription factor. The expression level of the target gene is assessed by monitoring luciferase expression which can be interpreted from the photon output. This approach has been widely use to study viral gene expression [56], oncogene regulation [57], heat shock genes [58,59], genes involved in circadian clock rhythms [60], and genes involved in inflammation and various disease states [61–67]. As an example, Keller and coworkers [67] investigated the expression of glial fibrillary acidic protein (GFAP) during the progression of amyotrophic lateral sclerosis (ALS). The authors generated a GFAP-luciferase reporter so that the regulation of GFAP could be visualized via bioluminescence output. Transgenic mice expressing the reporter construct were continuously imaged during the progression of ALS. Their findings demonstrated that GFAP induction in Schwann cells signified an onset of ALS. BLI facilitates the visualization of critical gene expression patterns in different stages of disease and advances the understanding of disease progression *in vivo*.

BLI has not only been used to monitor endogenous gene expression, but also has been widely utilized to visualize transgene delivery and expression *in vivo* since it is fast, sensitive, and noninvasive. Efficacy of gene transfer is evaluated by monitoring bioluminescent readout in the same living subject repeatedly over time without sacrificing animals. In recent years, investigators have employed BLI to assess many viral- and non viral-mediated gene transfer protocols [68–72].

### 7.4. Evaluating Protein Stability and Interaction

BLI benefits not only studies of monitoring gene expression at the transcriptional level, but also assessing biological processes at the level of protein function and interaction. One method to evaluate protein expression and stability is to fuse a reporter protein (usually FLuc) to the protein of interest. The stability of the target protein can be monitored by the bioluminescent output from the luciferase function. Temporal changes in signal intensity tell investigators the dynamics of abundance of the protein in question. For example, Lehmann *et al*. [61] constructed a fusion protein containing HIF-1α and firefly luciferase to study the stabilization of HIF-1α in tumor development *in vivo*. HIFs (hypoxia inducible factors) regulate genes involved in cellular response to hypoxia and play a critical role in cancer biology [73]. It has been demonstrated that HIF-1α is more abundant in some tumor cells than in normal cells [74]. In this study, a murine colon cancer cell line, C51, was stably transfected with the fusion reporter and subcutaneously injected into nude mice to create an allograft model. BLI was used to measure total photon flux in HIF-1α-Fluc-expressing tumors as the tumors grew. Their results revealed an increase in HIF-1α level in the early phase of tumor development and a dramatic decrease when the tumor volume was up to 1 cm^3^. The HIF signaling pathway has become an attractive target for anticancer treatment [73]. The BLI allograft model constructed in this study will assist drug development by providing a tool to visualize the efficacy of drugs in regulating HIF targets *in vivo*.

In addition to monitoring the stability of a given protein, investigators also use BLI to assess the activities of general protein degradation machinery. In one example of such work, Luker *et al*. [75] generated a ubiquitin-luciferase fusion reporter to monitor the activity of 26S proteasome *in vivo* by assessing the degradation of the reporter. This change in activity was thus represented by the changes in bioluminescent output. A similar application of BLI has been its use in reporter complementation assays, which have been widely used to assess protease activities [75–78]. In such cases, split fragments of luciferase are separated by a linker sequence that is recognized as a substrate by the particular protease of interest. In the presence of target enzyme, cleavage of the linker allows the split fragments to re-associate back to a fully functional protein and produce a bioluminescent signal. Recently, Wang and coworkers [78] used this approach to noninvasively monitor the activity of Hepatitis C virus (HCV) NS3/4A serine protease which is essential for viral reproduction *in vivo*. The reporter was constructed by separating the N-terminus and C-terminus of firefly luciferase and fusing them to interacting peptides (peptide A and peptide B), respectively, with NS3/4A cleavage sites. The reporter plasmid was co-injected with a plasmid (pNS3/4A) encoding the HCV NS3/4A protease sequence or a control plasmid into living mice to validate the reporter *in vivo*. BLI revealed an increase in bioluminescent output in mice co-injected with pNS3/4A compared to mice co-injected with a vehicle control plasmid. Moreover, the authors demonstrated the ability of this reporter to screen NS3/4A inhibitors in living animals.

Many biological events involve protein-protein interactions that can be affected by various physiological conditions. Traditionally, protein interactions were studied by means of a two-hybrid system in yeast. However, complete understanding of protein interactions requires assessing the subjects within relevant cellular microenvironments. BLI allows *in vivo* visualization of protein interactions as they happen in living animals in real time. In such cases, luciferase is split into two non-functional fragments (NLuc and CLuc), each of which is fused to one of the two proteins of interest. Interaction between query proteins brings NLuc and CLuc fragments close to each other to form a fully functional luciferase. When the split reporter is introduced into living animals, bioluminescent output can be read as an indicator of protein interactions *in vivo*. Paulmurugan *et al*. [24] for the first time demonstrated the application of split firefly luciferase complementation BLI to image MyoD-Id interaction in living mice. Later, the same group generated a split synthetic Renilla luciferase complementation assay to image drug-modulated heterodimerization of two human proteins *in vivo* [79]. Recently, Luker and colleagues [80] successfully utilized this approach to image activation and inhibition of chemokine receptor CXCR4 signaling in breast cancer metastasis *in vivo* by detecting interactions between CXCR4 and β-arrestin. This study established a new imaging model to probe CXCR signaling pathways and to screen for inhibitors in living animals.

## 8. Recent Advances

It is no surprise that with increasing interest and publication rates, more investigators are becoming involved in whole animal BLI research. With the increased demand for improved techniques and technologies comes the advances that move the field forward. One of the long standing problems has been the necessity for repeated injection of a substrate compound when FLuc or RLuc are employed as target reporter proteins. In order to reduce the amount of sequential injections required to illicit bioluminescent output, a method has recently been adopted that encapsulates the D-luciferin substrate of FLuc into a liposome, which can then be tailored to release the substrate at either a rapid, or gradually increasing rate. Intratumoral injections of quick-release liposomes allowed for an initial burst of detectable light, while intravenous injection of slow-release liposomes lead to a slow increase in radiance over 4–7 hours [141]. This choice of fast or prolonged luciferin release can provide researchers with customizable options to reduce the strain of repeated substrate injection on their animal models.

To alleviate the potential resolution problems associated with imaging small metastatic tumor formation in living tissues, it has recently been demonstrated that the naturally secreted Gaussia luciferase (GLuc) protein can be used as a proxy for overall tumor burden. When tumor cells are tagged with the gene driving production of GLuc, the resulting protein product will be secreted into the bloodstream where it can then be imaged and subsequently correlated to overall tumor cell prevalence. In addition to demonstrating the feasibility of using GLuc to track system-wide metastatic prevalence, it can also be used to continuously monitor for treatment response without the need to isolate and image individual areas [142], making it an excellent proxy for quickly evaluating the effectiveness of anticancer compounds over time *in vivo*.

**Figure 1.**
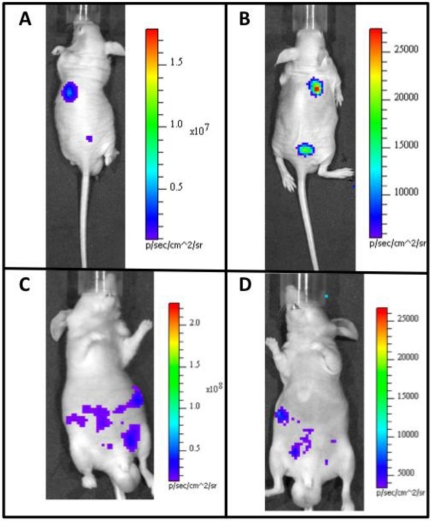
*In vivo* comparison of the FLuc and Lux reporter systems in a mouse model. Following subcutaneous injection of both **(A)** FLuc-tagged cells and luciferin or **(B)** Lux-tagged cells alone, the subject is imaged to determine the size and placement of the target cellular population within the animal. Similar experiments can be performed for **(C)** FLuc or **(D)** Lux-tagged cells following intraperitoneal injection. Although the average radiance of the FLuc cells is greater than that of Lux cells (note inset scale values), the low background detection ofsmall animal BLI allows for similar localization of cellular populations within the subject.

There has also been recent success in adapting the Lux system for autonomous function in mammalian cells (Figure 1). The Lux system is unique because it is capable of synthesizing all of its required substrate components from endogenously available cellular components. This circumvents the problems associated with differential injection routes and the dynamic luminescent expression profiles associated with repeated substrate injection using alternate luciferase systems. It has now been shown that mammalian codon-optimized *lux* genes can be expressed in mammalian cells and produce detectable bioluminescent signals at a wavelength of 490 nm over periods of days when constitutively induced. When mammalian cells expressing bioluminescent signal from the *lux* genes are subcutaneously injected into small animal models, they are able to function as tumor mimics that can combine the substrate-less detection characteristics of fluorescent reporters with the low background levels of bioluminescent reporter systems [119]. The ability to perform whole animal BLI without exogenous substrate addition will open the door for continuous, real-time imaging of animal subjects and provide investigators with a new luciferase for multiple reporter studies, increasing the usefulness of this technique. Because of the reagentless nature of Lux expression, it can easily be used in conjunction with existing reporter systems, prior to or following injection of the requisite substrate or introduction of an excitation wavelength of the chosen co-reporter system(s). In addition, the lack of a dynamic bioluminescent production rate in response to substrate addition allows the target populations of cells to be correlated to bioluminescent output at any time point during an experiment [119].

## 9. Conclusions

In the relatively short period of time since its introduction, whole animal BLI has become an invaluable technique for the noninvasive monitoring of small animal subjects that has yielded invaluable contributions to a variety of scientific fields. The majority of BLI experiments take advantage of the well-characterized FLuc or RLuc luciferase proteins, and when used in conjunction with alternate imaging technologies, they can provide extremely thorough and sophisticated datasets. However, one must take care to select the appropriate route of substrate injection upon supplementation of their associated substrate compounds. Despite the shortcomings of the currently available luciferase systems, they can often be adapted to provide information that would previously remain hidden from view, and recent advances in the field that can increase detection of small tumors, improve regulation of substrate availability, or negate it entirely make the future of BLI bright indeed.

## Figures and Tables

**Table 1.** Comparison of BLI reporter proteins.

**Reporter**	**Advantages**	**Disadvantages**
Firefly and click beetle luciferase D-luciferin substrate	High sensitivity and low signal-to-noise ratio Quantitative correlation between signal strength and cell numbers Low background in animal tissues Variations of firefly luciferase (stabilized and red-shifted) and click beetle luciferases (red and green) are available Different colors allow multi-component monitoring	Requires exogenous luciferin addition Fast consumption of luciferin can lead to unstable signal ATP and oxygen dependent Currently not practical for large animal models
Renilla and Gaussia luciferase Coelenterazine substrate	High sensitivity Quantitative correlation between signal strength and cell numbers Stabilized and red-shifted Renilla luciferase are available Secretion of Gaussia luciferase allows for subject-independent bioluminescence measurement	Requires exogenous coelenterazine addition Low anatomic resolution Increased background due to oxidation of coelenterazine by serum Oxygen dependent Fast consumption of coelenterazine can lead to unstable signal Currently not practical for large animal models
Bacterial luciferase	High sensitivity and low signal-to-noise ratio Quantitative correlation between signal strength and cell numbers Fully autonomous system, no requirement for addition of exogenous substrate Noninvasive Stable signal Rapid detection permitting real-time monitoring	Bioluminescence at 490 nm prone to absorptionin animal tissues Low anatomic resolution NADPH and oxygen dependent Not as bright as other luciferases Currently not practical for large animal models

**Table 2.** Commercial manufacturers of *in vivo* imaging systems.

**Company**	**URL**
Caliper Life Sciences	http://www.caliperls.com/tech/optical-imaging/
Berthold Technologies	http://www.berthold.com/ww/en/pub/home.cfm
Carestream	http://www.carestreamhealth.com/in-vivo-imaging-systems.html
Photometrics	http://www.photometrics.com/
Li-Cor Biosciences	http://www.licor.com/index.jsp
Cambridge Research & Instrumentation	http://www.cri-inc.com/index.asp
UVP	http://www.uvp.com/

**Table 3.** Selected BLI applications of firefly and Renilla luciferases (FLuc and RLuc).

**Applications**	**Examples**	**References**
Cell trafficking (survival, proliferation, migration, and function) in living animals	Stem cells (SCs), such as hematopoietic SCs, embryonic SCs, mesenchymal SCs, bone marrow mononuclear cells, and muscle SCs	[33–41,49,50,81,82]
	Immune effector cells such as cytokine-induced killer cells and NK-T cells	[42,43,45,51,53,55]
	Transplanted tissues	[46–48]

Noninvasive imaging of tumor development	Tumor growth, metastasis, and response to therapies	[25,55,83–90]

*In vivo* imaging of gene expression (conditional, spatial, and temporal patterns)	*In vivo* control of HIV promoter	[56]
	HIF-1 transcriptional activity in tumor hypoxia	[61,62,73,91,92]
	Hsp70 expression during heat shock and laser irradiation	[58,59,93,94]
	Cox-2 gene expression	[63, 64]
	Hes1-Luc expression to assess somite segmentation clock	[95]
	P53 expression and screening for antitumor compounds	[96]
	Per2 expression in CNS circadian clock	[60]
	TGF-β transcriptional activity in breast cancer bone metastasis	[65]
	GFAP expression in neurological disease	[66,67,97,98]
	HO-1 expression in hepatic ischemia	[99]
	TLR2 response in brain injury and inflammation	[100]
	MYC oncogene inactivation in liver cancer	[57]
	Smad signaling in injury and neurodegeneration	[101,102]

Evaluation of gene therapy (gene transfer and expression after delivery) in living animals	*In vivo* imaging of hydrodynamically dosed gene transfer	[68]
	*In utero* delivery of adeno-associated viral vectors	[69,103,104]
	Plasmid-mediated gene therapy for muscular dystrophy	[70]
	siRNA-mediated gene silencing	[72]

Real-time, *in vivo* monitoring of inflammation and infection	Viral infection and evaluation of virus vaccines	[105–109]
	Parasitic and fungal infections	[110–114]
	Biomaterial-associated infection	[115]

Monitoring protein-protein interaction in living animals	CXCR4 and β-arrestin interaction in breast cancer	[80]
	MyoD-Id protein interaction in living mice	[24]
	Rapamycin-modulated dimerization of two proteins	[79]
	Gal4-FLuc transgenic mice as universal reporters for protein-protein interaction (e.g., p53 and large T antigen)	[116]

Monitoring protein stability and function *in vivo*	Complementation Luc reporter to detect caspase-3 activity and monitoring of apoptosis	[76,77]
	Proteasome activity and proteasome inhibitor screening	[75]
	Furin (an endoprotease) activity in breast cancer	[117]
	Complementation Luc assay to detect hepatitis C virus NS3/4A serine protease activity *in vivo*	[78]
	Complementation Luc assay to assess HIF-1α stability and function in tumor hypoxia	[118]

**Table 4.** Selected BLI applications of bacterial luciferase (Lux).

**Organism**	**Application**	**References**
Human embryonic kidney (HEK293) cells	Whole animal imaging	[119]

*Escherichia coli*	Detection of *E. coli* O157:H7 in food and water	[120]
	*In vivo* imaging of *E. coli* colonization in mice	[121]
	Screening for interaction between antibiotics	[122]
	*In vivo* imaging of *E. coli* in wound infections	[123]

*Salmonella*	Monitoring the role of nitric oxide in tumor therapy	[124]
	Noninvasive imaging of *Salmonella* invasion	[125–128]
	Testing the susceptibility of neonate to vaccine	[129]

*Pseudomonas aeruginosa*	*In vivo* imaging of *P. aeruginosa* wound infection and evaluation of treatment	[130]

*Streptococcus pneumoniae*	Real-time monitoring of the pharmacodynamics of gemifloxacin	[131]
	Monitoring pneumococcal infection in the lungs of live mice	[132]

*Staphylococcus aureus*	Monitoring of *S. aureus* infection in living mice	[133]
	Noninvasive monitoring of bacterial contamination on biomaterial surfaces and the related immune response	[134]
	Assessing efficacy of antibiotics against bacterial biofilm formation in live mice	[135]
	Visualization of intracellular *S. aureus* replication and response to antibiotic treatment	[136]
*Listeria monocytogenes*	Monitoring infection over time, visualization of bone narrow as a niche for *L. monocytogenes* during the latent period	[137]

*Bifidobacterium breve* UCC2003	Tracking *Bifidobacterium* in mice *in vivo*	[138]

*Bacillus anthracis*	*In vivo* monitoring of *B. anthracis* spore germination in mice	[139]

*Yersinia enterocolitica*	*In vivo* assessment of *Y. enterocolitica* colonization and infection	[140]

## References

[1] Baker M. (2010). The whole picture. Nature.

[2] Wood K.V., Lam Y.A., Seliger H.H., McElroy W.D. (1989). Complementary DNA coding click beetle luciferase can elicit bioluminescence of different colors. Science.

[3] Lorenz W.W., McCann R.O., Longiaru M., Cormier M.J. (1991). Isolation and expression of a cDNA-encoding *Renilla reniformis* luciferase. Proc. Natl. Acad. Sci. USA.

[4] Verhaegen M., Christopoulos T.K. (2002). Recombinant Gaussia luciferase. Overexpression, purification, and analytical application of a bioluminescent reporter for DNA hybridization. Anal. Chem.

[5] Markova S.V., Golz S., Frank L.A., Kalthof B., Vysotski E.S. (2004). Cloning and expression of cDNA for a luciferase from the marine copepod *Metridia longa*—A novel secreted bioluminescent reporter enzyme. J. Biol. Chem.

[6] Thompson E.M., Nagata S., Tsuji F.I. (1989). Cloning and expression of cDNA for the luciferase from the marine ostracod *Vargula hilgendorfii*. Proc. Natl. Acad. Sci. USA.

[7] Meighen E.A. (1991). Molecular biology of bacterial bioluminescence. Microbiol. Rev.

[8] Fraga H. (2008). Firefly luminescence: A historical perspective and recent developments. Photochem. Photobiol. Sci.

[9] Conti E., Franks N.P., Brick P. (1996). Crystal structure of firefly luciferase throws light on a superfamily of adenylate-forming enzymes. Structure.

[10] Ando Y., Niwa K., Yamada N., Enomot T., Irie T., Kubota H., Ohmiya Y., Akiyama H. (2008). Firefly bioluminescence quantum yield and colour change by pH-sensitive green emission. Nat. Photonics.

[11] Gould S.J., Subramani S. (1988). Firefly luciferase as a tool in molecular and cell biology. Anal. Biochem.

[12] Close D.M., Ripp S., Sayler G.S. (2009). Reporter proteins in whole-cell optical bioreporter detection systems, biosensor integrations, and biosensing applications. Sensors.

[13] Troy T., Jekic-McMullen D., Sambucetti L., Rice B. (2004). Quantitative comparison of the sensitivity of detection of fluorescent and bioluminescent reporters in animal models. Mol. Imaging.

[14] Chance B., Cope M., Gratton E., Ramanujam N., Tromberg B. (1998). Phase measurement of light absorption and scatter in human tissue. Rev. Sci. Instrum.

[15] Vijayaraghavn R., Islam S.K., Zhang M., Ripp S., Caylor S., Weathers B., Moser S., Terry S., Blalock B., Sayler G.S. (2007). A bioreporter bioluminescent integrated circuit for very low-level chemical sensing in both gas and liquid environments. Sens. Actuat. B-Chem.

[16] Vaupel P., Kallinowski F., Okunieff P. (1989). Blood-flow, oxygen and nutrient supply, and metabolic microenvironment of human tumors—A review. Cancer Res.

[17] Kim J.B., Urban K., Cochran E., Lee S., Ang A., Rice B., Bata A., Campbell K., Coffee R., Gorodinsky A., Lu Z., Zhou H., Kishimoto T.K., Lassota P. (2010). Non-invasive detection of a small number of bioluminescent cancer cells *in vivo*. PLoS One.

[18] McMillin D.W., Delmore J., Weisberg E., Negri J.M., Geer D.C., Klippel S., Mitsiades N., Schlossman R.L., Munshi N.C., Kung A.L., Griffin J.D., Richardson P.G., Anderson K.C., Mitsiades C.S. (2010). Tumor cell-specific bioluminescence platform to identify stroma-induced changes to anticancer drug activity. Nat. Med.

[19] Honma S., Yoshikawa T., Nishide S., Ono D., Honma K., Tamaki N., Kuge Y. (2010). Bioluminescent imaging for assessing heterogeneous cell functions in the mammalian central circadian clock. Molecular Imaging for Integrated Medical Therapy and Drug Development.

[20] Heikkila J.E., Vaha-Koskela M.J.V., Ruotsalainen J.J., Martikainen M.W., Stanford M.M., McCart J.A., Bell J.C., Hinkkanen A.E. (2010). Intravenously administered alphavirus vector VA7 eradicates orthotopic human glioma xenografts in nude mice. PLoS One.

[21] Audigier S., Guiramand J., Prado-Lourenco L., Conte C., Gonzalez-Herrera I.G., Cohen-Solal C., Recasens M., Prats A.C. (2008). Potent activation of FGF-2 IRES-dependent mechanism of translation during brain development. RNA-Publ. RNA Soc.

[22] Ward W.W., Cormier M.J. (1978). Energy transfer via protein-protein interaction in Renilla bioluminescence. Photochem. Photobiol.

[23] Angers S., Salahpour A., Joly E., Hilairet S., Chelsky D., Dennis M., Bouvier M. (2000). Detection of B_2_-adrenergic receptor dimerization in living cells using bioluminescence resonance energy transfer (BRET). Proc. Natl. Acad. Sci. USA.

[24] Paulmurugan R., Umezawa Y., Gambhir S.S. (2002). Noninvasive imaging of protein-protein interactions in living subjects by using reporter protein complementation and reconstitution strategies. Proc. Natl. Acad. Sci. USA.

[25] Deroose C.M., De A., Loening A.M., Chow P.L., Ray P., Chatziioannou A.F., Gambhir S.S. (2007). Multimodality imaging of tumor xenografts and metastases in mice with combined small-animal PET, small-animal CT, and bioluminescence imaging. J. Nucl. Med.

[26] Inoue Y., Kiryu S., Izawa K., Watanabe M., Tojo A., Ohtomo K. (2009). Comparison of subcutaneous and intraperitoneal injection of D-luciferin for *in vivo* bioluminescence imaging. Eur. J. Nucl. Med. Mol. Imaging.

[27] Keyaerts M., Verschueren J., Bos T.J., Tchouate-Gainkam L.O., Peleman C., Breckpot K., Vanhove C., Caveliers V., Bossuyt A., Lahoutte T. (2008). Dynamic bioluminescence imaging for quantitative tumour burden assessment using IV or IP administration of D-luciferin: Effect on intensity, time kinetics and repeatability of photon emission. Eur. J. Nucl. Med. Mol. Imaging.

[28] Baba S., Cho S.Y., Ye Z., Cheng L., Engles J.M., Wahl R.L. (2007). How reproducible is bioluminescent imaging of tumor cell growth? Single time point *versus* the dynamic measurement approach. Mol. Imaging.

[29] Lee K.H., Byun S.S., Paik J.Y., Lee S.Y., Song S.H., Choe Y.S., Kim B.T. (2003). Cell uptake and tissue distribution of radioiodine labelled D-luciferin: Implications for luciferase based gene imaging. Nucl. Med. Commun.

[30] Bryant M.J., Chuah T.L., Luff J., Lavin M.F., Walker D.G. (2008). A novel rat model for glioblastoma multiforme using a bioluminescent F98 cell line. J. Clin. Neurosci.

[31] Zhang C., Yan Z.M., Arango M.E., Painter C.L., Anderes K. (2009). Advancing bioluminescence imaging technology for the evaluation of anticancer agents in the MDA-MB-435-HAL-Luc mammary fat pad and subrenal capsule tumor models. Clin. Cancer Res.

[32] Virostko J., Radhika A., Poffenberger G., Chen Z.Y., Brissova M., Gilchrist J., Coleman B., Gannon M., Jansen E.D., Powers A.C. (2010). Bioluminescence imaging in mouse models quantifies beta cell mass in the pancreas and after islet transplantation. Mol. Imaging Biol.

[33] Barberi T., Bradbury M., Dincer Z., Panagiotakos G., Socci N.D., Studer L. (2007). Derivation of engraftable skeletal myoblasts from human embryonic stem cells. Nat. Med.

[34] Cao F., Lin S., Xie X.Y., Ray P., Patel M., Zhang X.Z., Drukker M., Dylla S.J., Connolly A.J., Chen X.Y., Weissman I.L., Gambhir S.S., Wu J.C. (2006). *In vivo* visualization of embryonic stem cell survival, proliferation, and migration after cardiac delivery. Circulation.

[35] Cao Y.A., Wagers A.J., Beilhack A., Dusich J., Bachmann M.H., Negrin R.S., Weissman I.L., Contag C.H. (2004). Shifting foci of hematopoiesis during reconstitution from single stem cells. Proc. Natl. Acad. Sci. USA.

[36] Duan Y.Y., Catana A., Meng Y., Yamamoto N., He S.Q., Gupta S., Gambhir S.S., Zerna M.A. (2007). Differentiation and enrichment of hepatocyte-like cells from human embryonic stem cells *in vitro* and *in vivo*. Stem Cells.

[37] Sacco A., Doyonnas R., Kraft P., Vitorovic S., Blau H.M. (2008). Self-renewal and expansion of single transplanted muscle stem cells. Nature.

[38] Sasportas L.S., Kasmieh R., Wakimoto H., Hingtgen S., van de Water J., Mohapatra G., Figueiredo J.L., Martuza R.L., Weissleder R., Shah K. (2009). Assessment of therapeutic efficacy and fate of engineered human mesenchymal stem cells for cancer therapy. Proc. Natl. Acad. Sci. USA.

[39] Sheikh A.Y., Lin S.A., Cao F., Cao Y., van der Bogt K.E.A., Chu P., Chang C.P., Contag C.H., Robbins R.C., Wu J.C. (2007). Molecular imaging of bone marrow mononuclear cell homing and engraftment in ischemic myocardium. Stem Cells.

[40] Togel F., Yang Y., Zhang P., Hu Z.M., Westenfelder C. (2008). Bioluminescence imaging to monitor the *in vivo* distribution of administered mesenchymal stem cells in acute kidney injury. Am. J. Physiol.-Renal Physiol.

[41] van der Bogt K.E.A., Sheikh A.Y., Schrepfer S., Hoyt G., Cao F., Ransohoff K.J., Swijnenburg R.J., Pearl J., Lee A., Fischbein M., Contag C.H., Robbins R.C., Wu J.C. (2008). Comparison of different adult stem cell types for treatment of myocardial ischemia. Circulation.

[42] Zeiser R., Nguyen V.H., Beilhack A., Buess M., Schulz S., Baker J., Contag C.H., Negrin R.S. (2006). Inhibition of CD4^+^CD25^+^ regulatory T-cell function by calcineurin-dependent interleukin-2 production. Blood.

[43] Zeiser R., Nguyen V.H., Hou J.Z., Beilhack A., Zambricki E., Buess M., Contag C.H., Negrin R.S. (2007). Early CD30 signaling is critical for adoptively transferred CD4^+^CD25^+^ regulatory T cells in prevention of acute graft-*versus*-host disease. Blood.

[44] Alsayed Y., Ngo H., Runnels J., Leleu X., Singha U.K., Pitsillides C.M., Spencer J.A., Kimlinger T., Ghobrial J.M., Jia X.Y., Lu G.W., Timm M., Kumar A., Cote D., Veilleux I., Hedin K.E., Roodman G.D., WitZig T.E., Kung A.L., Hideshima T., Anderson K.C., Lin C.P., Ghobrial I.M. (2007). Mechanisms of regulation of CXCR4/SDF-1 (CXCL12)-dependent migration and homing in multiple myeloma. Blood.

[45] Dobrenkov K., Olszewska M., Likar Y., Shenker L., Gunset G., Cai S., Pillarsetty N., Hricak H., Sadelain M., Ponomarev V. (2008). Monitoring the efficacy of adoptively transferred prostate cancer-targeted human T lymphocytes with PET and bioluminescence imaging. J. Nucl. Med.

[46] Chen C.H., Yeh Y.C., Wu G.J., Huang Y.H., Lai W.F.T., Liu J.Y., Tzeng C.R. (2010). Tracking the rejection and survival of mouse ovarian iso- and allografts *in vivo* with bioluminescent imaging. Reproduction.

[47] Chen X.J., Zhang X.M., Larson C.S., Baker M.S., Kaufman D.B. (2006). *In vivo* bioluminescence imaging of transplanted islets and early detection of graft rejection. Transplantation.

[48] Cao Y.A., Bachmann M.H., Beilhack A., Yang Y., Tanaka M., Swijnenburg R.J., Reeves R., Taylor-Edwards C., Schulz S., Doyle T.C., Fathman C.G., Robbins R.C., Herzenberg L.A., Negrin R.S., Contag C.H. (2005). Molecular imaging using labeled donor tissues reveals patterns of engraftment, rejection, and survival in transplantation. Transplantation.

[49] Li Z.J., Lee A., Huang M., Chun H., Chung J., Chu P., Hoyt G., Yang P., Rosenberg J., Robbins R.C., Wu J.C. (2009). Imaging survival and function of transplanted cardiac resident stem cells. J. Am. Coll. Cardiol.

[50] van der Bogt K.E.A., Schrepfer S., Yu J., Sheikh A.Y., Hoyt G., Govaert J.A., Velotta J.B., Contag C.H., Robbins R.C., Wu J.C. (2009). Comparison of transplantation of adipose tissue- and bone marrow-derived mesenchymal stem cells in the infarcted heart. Transplantation.

[51] Hardy J., Edinger M., Bachmann M.H., Negrin R.S., Fathman C.G., Contag C.H. (2001). Bioluminescence imaging of lymphocyte trafficking *in vivo*. Exp. Hematol.

[52] Negrin R.S., Contag C.H. (2006). Innovation—*In vivo* imaging using bioluminescence: A tool for probing graft-*versus*-host disease. Nat. Rev. Immunol.

[53] Edinger M., Cao Y.A., Verneris M.R., Bachmann M.H., Contag C.H., Negrin R.S. (2003). Revealing lymphoma growth and the efficacy of immune cell therapies usingin *in vivo* bioluminescence imaging. Blood.

[54] Gao J.Q., Okada N., Mayumi T., Nakagawa S. (2008). Immune cell recruitment and cell-based system for cancer therapy. Pharm. Res.

[55] Edinger M., Hoffmann P., Contag C.H., Negrin R.S. (2003). Evaluation of effector cell fate and function by *in vivo* bioluminescence imaging. Methods.

[56] Contag C.H., Spilman S.D., Contag P.R., Oshiro M., Eames B., Dennery P., Stevenson D.K., Benaron D.A. (1997). Visualizing gene expression in living mammals using a bioluminescent reporter. Photochem. Photobiol.

[57] Shachaf C.M., Kopelman A.M., Arvanitis C., Karlsson A., Beer S., Mandl S., Bachmann M.H., Borowsky A.D., Ruebner B., Cardiff R.D., Yang Q.W., Bishop J.M., Contag C.H., Felsher D.W. (2004). MYC inactivation uncovers pluripotent differentiation and tumour dormancy in hepatocellular cancer. Nature.

[58] O’Connell-Rodwell C.E., Mackanos M.A., Simanovskii D., Cao Y.A., Bachmann M.H., Schwettman H.A., Contag C.H. (2008). *In vivo* analysis of heat-shock-protein-70 induction following pulsed laser irradiation in a transgenic reporter mouse. J. Biomed. Opt.

[59] Beckham J.T., Wilmink G.J., Mackanos M.A., Takahashi K., Contag C.H., Takahashi T., Jansen E.D. (2008). Role of HSP70 in cellular thermotolerance. Lasers Surg. Med.

[60] Liu A.C., Welsh D.K., Ko C.H., Tran H.G., Zhang E.E., Priest A.A., Buhr E.D., Singer O., Meeker K., Verma I.M., Doyle F.J., Takahashi J.S., Kay S.A. (2007). Intercellular coupling confers robustness against mutations in the SCN circadian clock network. Cell.

[61] Lehmann S., Stiehl D.P., Honer M., Dominietto M., Keist R., Kotevic I., Wollenick K., Ametamey S., Wenger R.H., Rudin M. (2009). Longitudinal and multimodalin *in vivo* imaging of tumor hypoxia and its downstream molecular events. Proc. Natl. Acad. Sci. USA.

[62] Harada H., Kizaka-Kondoh S., Li G., Itasaka S., Shibuya K., Inoue M., Hiraoka M. (2007). Significance of HIF-1-active cells in angiogenesis and radioresistance. Oncogene.

[63] Ishikawa T., Jain N., Herschman H.R. (2009). Feedback regulation of cyclooxygenase-2 transcription *ex vivo* and *in vivo*. Biochem. Biophys. Res. Commun.

[64] Ishikawa T.O., Jain N.K., Taketo M.M., Herschman H.R. (2006). Imaging cyclooxygenase-2 (Cox-2) gene expression in living animals with a luciferase knock-in reporter gene. Mol. Imaging. Biol.

[65] Korpal M., Yan J., Lu X., Xu S.W., Lerit D.A., Kang Y.B. (2009). Imaging transforming growth factor-beta signaling dynamics and therapeutic response in breast cancer bone metastasis. Nat. Med.

[66] Tamguney G., Francis K.P., Giles K., Lemus A., DeArmond S.J., Prusiner S.B. (2009). Measuring prions by bioluminescence imaging. Proc. Natl. Acad. Sci. USA.

[67] Keller A.F., Gravel M., Kriz J. (2009). Live imaging of amyotrophic lateral sclerosis pathogenesis: Disease onset is characterized by marked induction of GFAP in Schwann cells. Glia.

[68] Rettig G.R., McAnuff M., Liu D.J., Kim J.S., Rice K.G. (2006). Quantitative bioluminescence imaging of transgene expression *in vivo*. Anal. Biochem.

[69] Lamfers M.L.M., Fulci G., Gianni D., Tang Y., Kurozumi K., Kaur B., Moeniralm S., Saeki Y., Carette J.E., Weissleder R., Vandertop W.P., van Beusechem V.W., Dirven C.M.F., Chiocca E.A. (2006). Cyclophosphamide increases transgene expression mediated by an oncolytic adenovirus in glioma-bearing mice monitored by bioluminescence imaging. Mol. Ther.

[70] Bertoni C., Jarrahian S., Wheeler T.M., Li Y.N., Olivares E.C., Calos M.P., Rando T.A. (2006). Enhancement of plasmid-mediated gene therapy for muscular dystrophy by directed plasmid integration. Proc. Natl. Acad. Sci. USA.

[71] Lipshutz G.S., Flebbe-Rehwaldt L., Gaensler K.M.L. (2000). Reexpression following readministration of an adenoviral vector in adult mice after initial *in utero* adenoviral administration. Mol. Ther.

[72] Bartlett D.W., Davis M.E. (2006). Insights into the kinetics of siRNA-mediated gene silencing from live-cell and live-animal bioluminescent imaging. Nucleic Acids Res.

[73] Kizaka-Kondoh S., Tanaka S., Harada H., Hiraoka M. (2009). The HIF-1-active microenvironment: An environmental target for cancer therapy. Adv. Drug Deliv. Rev.

[74] Zhong H., De Marzo A.M., Laughner E., Lim M., Hilton D.A., Zagzag D., Buechler P., Isaacs W.B., Semenza G.L., Simons J.W. (1999). Overexpression of hypoxia-inducible factor 1 alpha in common human cancers and their metastases. Cancer Res.

[75] Luker G.D., Pica C.M., Song J.L., Luker K.E., Piwnica-Worms D. (2003). Imaging 26S proteasome activity and inhibition in living mice. Nat. Med.

[76] Coppola J.M., Ross B.D., Rehemtulla A. (2008). Noninvasive imaging of apoptosis and its application in cancer therapeutics. Clin. Cancer Res.

[77] Laxman B., Hall D.E., Bhojani M.S., Hamstra D.A., Chenevert T.L., Ross B.D., Rehemtulla A. (2002). Noninvasive real-time imaging of apoptosis. Proc. Natl. Acad. Sci. USA.

[78] Wang L.C., Fu Q.X., Dong Y.F., Zhou Y., Jia S.Z., Du J.A., Zhao F., Wang Y.L., Wang X.H., Peng J.C., Yang S.H., Zhan L.S. (2010). Bioluminescence imaging of hepatitis C virus NS3/4A serine protease activity in cells and living animals. Antivir. Res.

[79] Paulmurugan R., Massoud T.F., Huang J., Gambhir S.S. (2004). Molecular imaging of drug-modulated protein-protein interactions in living subjects. Cancer Res.

[80] Luker K.E., Gupta M., Luker G.D. (2008). Imaging CXCR4 signaling with firefly luciferase complementation. Anal. Chem.

[81] Gerdoni E., Gallo B., Casazza S., Musio S., Bonanni I., Pedemonte E., Mantegazza R., Frassoni F., Mancardi G., Pedotti R., Uccelli A. (2007). Mesenchymal stem cells effectively modulate pathogenic immune response in experimental autoimmune encephalomyelitis. Ann. Neurol.

[82] Lee S.W., Padmanabhan P., Ray P., Gambhir S.S., Doyle T., Contag C., Goodman S.B., Biswal S. (2009). Stem cell-mediated accelerated bone healing observed with *in vivo* molecular and small animal imaging technologies in a model of skeletal injury. J. Orthop. Res.

[83] Buschow C., Charo J., Anders K., Loddenkemper C., Jukica A., Alsamah W., Perez C., Willimsky G., Blankenstein T. (2010). *In vivo* imaging of an inducible oncogenic tumor antigen visualizes tumor progression and predicts CTL tolerance. J. Immunol.

[84] Hashizume R., Ozawa T., Dinca E.B., Banerjee A., Prados M.D., James C.D., Gupta N. (2010). A human brainstem glioma xenograft model enabled for bioluminescence imaging. J. Neurooncol.

[85] Lee C.J., Spalding A.C., Ben-Josef E., Wang L.D., Simeone D.M. (2010). *In vivo* bioluminescent imaging of irradiated orthotopic pancreatic cancer xenografts in NOD-SCID mice: A novel method for targeting and assaying efficacy of ionizing radiation. Transl. Oncol.

[86] Mezzanotte L., Fazzina R., Michelini E., Tonelli R., Pession A., Branchini B., Roda A. (2010). *In vivo* bioluminescence imaging of murine xenograft cancer models with a red-shifted thermostable luciferase. Mol. Imaging Biol.

[87] Negrin R.S., Edinger M., Verneris M., Cao Y.A., Bachmann M., Contag C.H. (2002). Visualization of tumor growth and response to NK-T cell based immunotherapy using bioluminescence. Ann. Hematol.

[88] Rehemtulla A., Stegman L.D., Cardozo S.J., Gupta S., Hall D.E., Contag C.H., Ross B.D. (2000). Rapid and quantitative assessment of cancer treatment response using *in vivo* bioluminescence imaging. Neoplasia.

[89] Sweeney T.J., Mailander V., Tucker A.A., Olomu A.B., Zhang W., Cao Y., Negrin R.S., Contag C.H. (1999). Visualizing the kinetics of tumor-cell clearance in living animals. Proc. Natl. Acad. Sci. USA.

[90] Venisnik K.M., Olafsen T., Loening A.M., Iyer M., Gambhir S.S., Wu A.M. (2006). Bifunctional antibody-Renilla luciferase fusion protein for *in vivo* optical detection of tumors. Protein Eng. Des. Sel.

[91] Harada H., Kizaka-Kondoh S., Hiraoka M. (2005). Optical imaging of tumor hypoxia and evaluation of efficacy of a hypoxia-targeting drug in living animals. Mol. Imaging.

[92] Viola R.J., Provenzale J.M., Li F., Li C.Y., Yuan H., Tashjian J., Dewhirst M.W. (2008). *In vivo* bioluminescence imaging monitoring of hypoxia-inducible factor 1 alpha, a promoter that protects cells, in response to chemotherapy. Am. J. Roentgenol.

[93] Beckham J.T., Mackanos M.A., Crooke C., Takahashi T., O’Connell-Rodwell C., Contag C.H., Jansen E.D. (2004). Assessment of cellular response to thermal laser injury through bioluminescence imaging of heat shock protein 70. Photochem. Photobiol.

[94] Wilmink G.J., Opalenik S.R., Beckham J.T., Mackanos M.A., Nanney L.B., Contag C.H., Davidson J.M., Jansen E.D. (2008). *In vivo* optical imaging of hsp70 expression to assess collateral tissue damage associated with infrared laser ablation of skin. J. Biomed. Opt.

[95] Masamizu Y., Ohtsuka T., Takashima Y., Nagahara H., Takenaka Y., Yoshikawa K., Okamura H., Kageyama R. (2006). Real-time imaging of the somite segmentation clock: Revelation of unstable oscillators in the individual presomitic mesoderm cells. Proc. Natl. Acad. Sci. USA.

[96] Wang W.G., Kim S.H., El-Deiry W.S. (2006). Small-molecule modulators of p53 family signaling and antitumor effects in p53-deficient human colon tumor xenografts. Proc. Natl. Acad. Sci. USA.

[97] Cordeau P., Lalancette-Hebert M., Weng Y.C., Kriz J. (2008). Live imaging of neuroinflammation reveals sex and estrogen effects on astrocyte response to ischemic injury. Stroke.

[98] Zhu L.Y., Ramboz S., Hewitt D., Boring L., Grass D.S., Purchio A.F. (2004). Non-invasive imaging of GFAP expression after neuronal damage in mice. Neurosci. Lett.

[99] Su H.W., van Dam G.M., Buis C.I., Visser D.S., Hesselink J.W., Schuurs T.A., Leuvenink H.G.D., Contag C.H., Porte R.J. (2006). Spatiotemporal expression of heme oxygenase-1 detected by *in vivo* bioluminescence after hepatic ischemia in HO-1/Luc mice. Liver Transplant.

[100] Lalancette-Hebert M., Phaneuf D., Soucy G., Weng Y.C., Kriz J. (2009). Live imaging of Toll-like receptor 2 response in cerebral ischaemia reveals a role of olfactory bulb microglia as modulators of inflammation. Brain.

[101] Lin A.H., Luo J., Mondshein L.H., ten Dijke P., Vivien D., Contag C.H., Wyss-Coray T. (2005). Global analysis of Smad2/3-dependent TGF-beta signaling in living mice reveals prominent tissue-specific responses to injury. J. Immunol.

[102] Luo J., Lin A.H., Masliah E., Wyss-Coray T. (2006). Bioluminescence imaging of Smad signaling in living mice shows correlation with excitotoxic neurodegeneration. Proc. Natl. Acad. Sci. USA.

[103] Lipshutz G.S., Gruber C.A., Cao Y.A., Hardy J., Contag C.H., Gaensler K.M.L. (2001). *In utero* delivery of adeno-associated viral vectors: Intraperitoneal gene transfer produces long-term expression. Mol. Ther.

[104] Lipshutz G.S., Titre D., Brindle M., Bisconte A.R., Contag C.H., Gaensler K.M.L. (2003). Comparison of gene expression after intraperitoneal delivery of AAV2 or AAV5 *in utero*. Mol. Ther.

[105] Cook S.H., Griffin D.E. (2003). Luciferase imaging of neurotropic viral infection in intact animals. J. Virol.

[106] Doyle T.C., Burns S.M., Contag C.H. (2004). *In vivo* bioluminescence imaging for integrated studies of infection. Cell Microbiol.

[107] Luker G.D., Bardill J.P., Prior J.L., Pica C.M., Piwnica-Worms D., Leib D.A. (2002). Noninvasive bioluminescence imaging of herpes simplex virus type 1 infection and therapy in living mice. J. Virol.

[108] Luker K.E., Hutchens M., Schultz T., Pekosz A., Luker G.D. (2005). Bioluminescence imaging of vaccinia virus: Effects of interferon on viral replication and spread. Virology.

[109] Luker K.E., Schujtz T., Joseph R., Leib D.A., Luker G.D. (2006). Transgenic reporter mouse for bioluminescence imaging of herpes simplex virus 1 infection in living mice. Virology.

[110] Doyle T.C., Nawotka K.A., Kawahara C.B., Francis K.P., Contag P.R. (2006). Visualizing fungal infections in living mice using bioluminescent pathogenic *Candida albicans* strains transformed with the firefly luciferase gene. Microb. Pathog.

[111] Franke-Fayard B., Janse C.J., Cunha-Rodrigues M., Ramesar J., Buscher P., Que I., Lowik C., Voshol P.J., den Boer M.A.M., van Duinen S.G., Febbraio M., Mota M.M., Waters A.P. (2005). Murine malaria parasite sequestration: CD36 is the major receptor, but cerebral pathology is unlinked to sequestration. Proc. Natl. Acad. Sci. USA.

[112] Hitziger N., Dellacasa I., Albiger B., Barragan A. (2005). Dissemination of *Toxoplasma gondii* to immunoprivileged organs and role of Toll/interleukin-1 receptor signalling for host resistance assessed by *in vivo* bioluminescence imaging. Cell Microbiol.

[113] Lang T., Goyard S., Lebastard M., Milon G. (2005). Bioluminescent *Leishmania* expressing luciferase for rapid and high throughput screening of drugs acting on amastigote-harbouring macrophages and for quantitative real-time monitoring of parasitism features in living mice. Cell Microbiol.

[114] Saeij J.P.J., Boyle J.P., Grigg M.E., Arrizabalaga G., Boothroyd J.C. (2005). Bioluminescence imaging of *Toxoplasma gondii* infection in living mice reveals dramatic differences between strains. Infect. Immun.

[115] Sjollema J., Sharma P.K., Dijkstra R.J.B., van Dam G.M., van der Mei H.C., Engelsman A.F., Busscher H.J. (2010). The potential for bio-optical imaging of biomaterial-associated infection *in vivo*. Biomaterials.

[116] Pichler A., Prior J.L., Luker G.D., Piwnica-Worms D. (2008). Generation of a highly inducible *Gal4* → *Fluc* universal reporter mouse for *in vivo* bioluminescence imaging. Proc. Natl. Acad. Sci. USA.

[117] Dragulescu-Andrasi A., Liang G.L., Rao J.H. (2009). *In vivo* bioluminescence imaging of furin activity in breast cancer cells using bioluminogenic substrates. Bioconjugate Chem.

[118] Moroz E., Carlin S., Dyomina K., Burke S., Thaler H.T., Blasberg R., Serganova I. (2009). Real-time imaging of HIF-1 alpha stabilization and degradation. PloS One.

[119] Close D.M., Patterson S.S., Ripp S., Baek S.J., Sanseverino J., Sayler G.S. (2010). Autonomous bioluminescent expression of the bacterial luciferase gene cassette (*lux*) in a mammalian cell line. PLoS One.

[120] Ripp S., Jegier P., Johnson C.M., Brigati J., Sayler G.S. (2008). Bacteriophage-amplified bioluminescent sensing of *Escherichia coli* O157:H7. Anal. Bioanal. Chem.

[121] Foucault M.L., Thomas L., Goussard S., Branchini B.R., Grillot-Courvalin C. (2010). *In vivo* bioluminescence imaging for the study of intestinal colonization by *Escherichia coli* in mice. Appl. Environ. Microbiol.

[122] Yeh P., Tschumi A.I., Kishony R. (2006). Functional classification of drugs by properties of their pairwise interactions. Nat. Genet.

[123] Hamblin M.R., O’Donnell D.A., Murthy N., Contag C.H., Hasan T. (2002). Rapid control of wound infections by targeted photodynamic therapy monitored by *in vivo* bioluminescence imaging. Photochem. Photobiol.

[124] Barak Y., Schreiber F., Thorne S.H., Contag C.H., deBeer D., Matin A. (2010). Role of nitric oxide in *Salmonella typhimurium*-mediated cancer cell killing. BMC Cancer.

[125] Burns-Guydish S.M., Olomu I.N., Zhao H., Wong R.J., Stevenson D.K., Contag C.H. (2005). Monitoring age-related susceptibility of young mice to oral *Salmonella enterica* serovar *typhimurium* infection using an *in vivo* murine model. Pediatr. Res.

[126] Contag C.H., Contag P.R., Mullins J.I., Spilman S.D., Stevenson D.K., Benaron D.A. (1995). Photonic detection of bacterial pathogens in a living host. Molec. Micro.

[127] Flentie K.N., Qi M., Gammon S.T., Razia Y., Lui F.L., Marpegan L., Manglik A., Piwnica-Worms D., McKinney J.S. (2008). Stably integrated *luxCDABE* for assessment of *Salmonella* invasion kinetics. Mol. Imaging.

[128] Moulton K., Ryan P., Lay D., Willard S. (2009). Postmortem photonic imaging of *lux*-modified *Salmonella* Typhimurium within the gastrointestinal tract of swine after oral inoculation *in vivo*. J. Anim. Sci.

[129] Burns-Guydish S.M., Zhao H., Stevenson D.K., Contag C.H. (2007). The potential *Salmonella aroA^−^* vaccine strain is safe and effective in young BALB/c mice. Neonatology.

[130] Hamblin M.R., Zahra T., Contag C.H., McManus A.T., Hasan T. (2003). Optical monitoring and treatment of potentially lethal wound infections *in vivo*. J. Infect. Dis.

[131] Beard S.J., Salisbury V., Lewis R.J., Sharpe J.A., MacGowan A.P. (2002). Expression of *lux* genes in a clinical isolate of *Streptococcus pneumoniae*: Using bioluminescence to monitor gemifloxacin activity. Antimicrob. Agents Chemother.

[132] Francis K.P., Yu J., Bellinger-Kawahara C., Joh D., Hawkinson M.J., Xiao G., Purchio T.F., Caparon M.G., Lipsitch M., Contag P.R. (2001). Visualizing pneumococcal infections in the lungs of live mice using bioluminescent *Streptococcus pneumoniae* transformed with a novel gram-positive *lux* transposon. Infect. Immun.

[133] Francis K.P., Joh D., Bellinger-Kawahara C., Hawkinson M.J., Purchio T.F., Contag P.R. (2000). Monitoring bioluminescent *Staphylococcus aureus* infections in living mice using a novel *luxABCDE* construct. Infect. Immun.

[134] Engelsman A.F., van der Mei H.C., Francis K.P., Busscher H.J., Ploeg R.J., van Dam G.M. (2009). Real time noninvasive monitoring of contaminating bacteria in a soft tissue implant infection model. J. Biomed. Mater. Res. B.

[135] Kadurugamuwa J.L., Sin L.V., Yu J., Francis K.P., Kimura R., Purchio T., Contag P.R. (2003). Rapid direct method for monitoring antibiotics in a mouse model of bacterial biofilm infection. Antimicrob. Agents Chemother.

[136] Qazi S.N.A., Harrison S.E., Self T., Williams P., Hill P.J. (2004). Real-time monitoring of intracellular *Staphylococcus aureus* replication. J. Bacteriol.

[137] Hardy J., Chu P., Contag C.H. (2009). Foci of *Listeria monocytogenes* persist in the bone marrow. Dis. Model. Mech.

[138] Cronin M., Sleator R.D., Hill C., Fitzgerald G.F., van Sinderen D. (2008). Development of a luciferase-based reporter system to monitor *Bifidobacterium breve* UCC2003 persistence in mice. BMC Microbiol.

[139] Sanz P., Teel L.D., Alem F., Carvalho H.M., Darnell S.C., O’Brien A.D. (2008). Detection of *Bacillus anthracis* spore germination *in vivo* by bioluminescence imaging. Infect. Immun.

[140] Trcek J., Berschl K., Trulzsch K. (2010). *In vivo* analysis of *Yersinia enterocolitica* infection using *luxCDABE*. FEMS Microbiol. Lett.

[141] Kheirolomoom A., Kruse D.E., Qin S.P., Watson K.E., Lai C.Y., Young L.J.T., Cardiff R.D., Ferrara K.W. (2010). Enhanced *in vivo* bioluminescence imaging using liposomal luciferin delivery system. J. Control. Release.

[142] Chung E., Yamashita H., Au P., Tannous B.A., Fukumura D., Jain R.K. (2009). Secreted Gaussia luciferase as a biomarker for monitoring tumor progression and treatment response of systemic metastases. PLoS One.

